# 1′-*O*-methyl-averantin isolated from the endolichenic fungus *Jackrogersella* sp. EL001672 suppresses colorectal cancer stemness via sonic Hedgehog and Notch signaling

**DOI:** 10.1038/s41598-023-28773-z

**Published:** 2023-02-16

**Authors:** Mücahit Varlı, Eun-Young Lee, Yi Yang, Rui Zhou, İsa Taş, Sultan Pulat, Chathurika D. B. Gamage, So-Yeon Park, Jae-Seoun Hur, Sang-Jip Nam, Hangun Kim

**Affiliations:** 1 College of Pharmacy, Sunchon National University, 255 Jungang-ro, Sunchon, Jeonnam 57922 Republic of Korea; 2 Department of Chemistry and Nanoscience, Ewha Womans University, Seoul, 03760 Republic of Korea; 3 Korean Lichen Res. Institute, Sunchon National University, 255 Jungang-ro, Sunchon, Jeonnam 57922 Republic of Korea

**Keywords:** Cancer stem cells, Phenotypic screening

## Abstract

Endolichenic fungi are host organisms that live on lichens and produce a wide variety of secondary metabolites. Colorectal cancer stem cells are capable of self-renewal and differentiation into cancer cells, which makes cancers difficult to eradicate. New alternative therapeutics are needed to inhibit the growth of tumor stem cells. This study examined the ability of an extract of *Jackrogersella* sp. EL001672 (derived from the lichen *Cetraria* sp.) and the isolated compound 1′-*O*-methyl-averantin to inhibit development of cancer stemness. The endolichenic fungus *Jackrogersella* sp. EL001672 (KACC 83021BP), derived from *Cetraria* sp., was grown in culture medium. The culture broth was extracted with acetone to obtain a crude extract. Column chromatography and reverse-phase HPLC were used to isolate an active compound. The anticancer activity of the extract and the isolated compound was evaluated by qRT-PCR and western blotting, and in cell viability, spheroid formation, and reporter assays. The acetone extract of EL001672 did not affect cell viability. However, 1′-*O*-methyl-averantin showed cytotoxic effects against cancer cell lines at 50 μg/mL and 25 μg/mL. Both the crude extract and 1′-*O*-methyl-averantin suppressed spheroid formation in CRC cell lines, and downregulated expression of stemness markers ALDH1, CD44, CD133, Lgr-5, Msi-1, and EphB1. To further characterize the mechanism underlying anti-stemness activity, we examined sonic Hedgehog and Notch signaling. The results showed that the crude extract and the 1′-*O*-methyl-averantin inhibited Gli1, Gli2, SMO, Bmi-1, Notch-1, Hes-1, and the CSL complex. Consequently, an acetone extract and 1′-*O*-methyl-averantin isolated from EL001672 suppresses colorectal cancer stemness by regulating the sonic Hedgehog and Notch signaling pathways.

## Introduction

Cancer stemness cells are known for their potential for self-renewal and their ability to initiate growth of heterogeneous cancer cells^1^. Increasing evidence shows that cancer stem cells (CSCs) contribute to chemoresistance^2,3^, which limits the efficacy of anticancer drugs^4^. CSCs are found in many different types of cancer, including brain, kidney, breast, blood, lung, pancreatic, prostate, and colon cancers^5,6^. Colon cancer is the second largest cause of cancer-related death, and the third most common cancer in the world in all sexes^7^. Although many therapeutic agents have been developed for the treatment of colon cancer, many treatments fail^4^. Therefore, it is important to prevent colon tumor formation and to search for new therapeutics that target CSCs.

Targeting CSCs primarily aims to block expression of stemness markers and embryonic signaling pathways (i.e., the sonic Hedgehog (SHH) and Notch pathways). CSCs express cluster of differentiation 133 (CD133), CD44, leucine-rich repeat-containing G protein-coupled receptor-5 (Lgr-5), and ephrin type-B receptor 1 (EPHB1)^8^, which are stem cell surface markers. In addition, ALDH1 is a stemness marker, overexpression of which correlates with development of CSCs^9^, and Musashi-1 promotes chemoresistance and cancer stemness in colorectal cancers^10^.

SHH is an important regulator of organogenesis during embryonic development^11^. SHH ligands cause degradation of PTCH1, which activates of the G-protein–coupled receptors Smoothened (SMO) and Gli Family Zinc Finger 2 (Gli2)^12,13^. In addition, inhibition of SMO regulates cell migration and cellular plasticity^14^. When acting as a transcription factor, Gli1 amplifies Hh signaling and so plays a key role in Hh signaling activity^15–17^. In another pathway, called the stem cell signaling pathway, Notch plays a role in critical processes such as breast development, normal hematopoiesis, and maturation of the colorectal epithelium^18,19^. Notch ligands are divided into two structurally separate groups, the Delta-like ligands (Dlls 1, 3, and 4) and the Jagged ligands (1 and 2), which bind to four transmembrane notch receptors^20^. Thus, targeted suppression of stem cell formation in colorectal cancer (and other malignancies), chemoresistance, and relapse requires specific interventions that target relevant pathways.

Lichens produce nearly 1050 specific secondary metabolites that have antimicrobial, antiviral, antioxidant, antiallergic, and antitumoral effects^3,21–27^. The Lichen thallus includes symbiotic endolichenic fungi within its structure. Endolichenic fungi species are thought to play an important ecological role by stimulating lichen formation and development, and by producing bioactive substances^28–32^. Here, we examined the ability of the known metabolite 1′-*O*-methyl-averantin^33,34^, isolated from a crude extract of the endolichenic fungus *Jackrogersella* sp. EL001672, to inhibit development of CSCs.

To the best of our knowledge, this is the first study to evaluate the anti-CSC potential of secondary metabolites of 1′-*O*-methyl-averantin from endolichenic fungus *Jackrogersella* sp.

## Results

### Identification of active compound from an EL001672 crude extract

**Figure 1:**
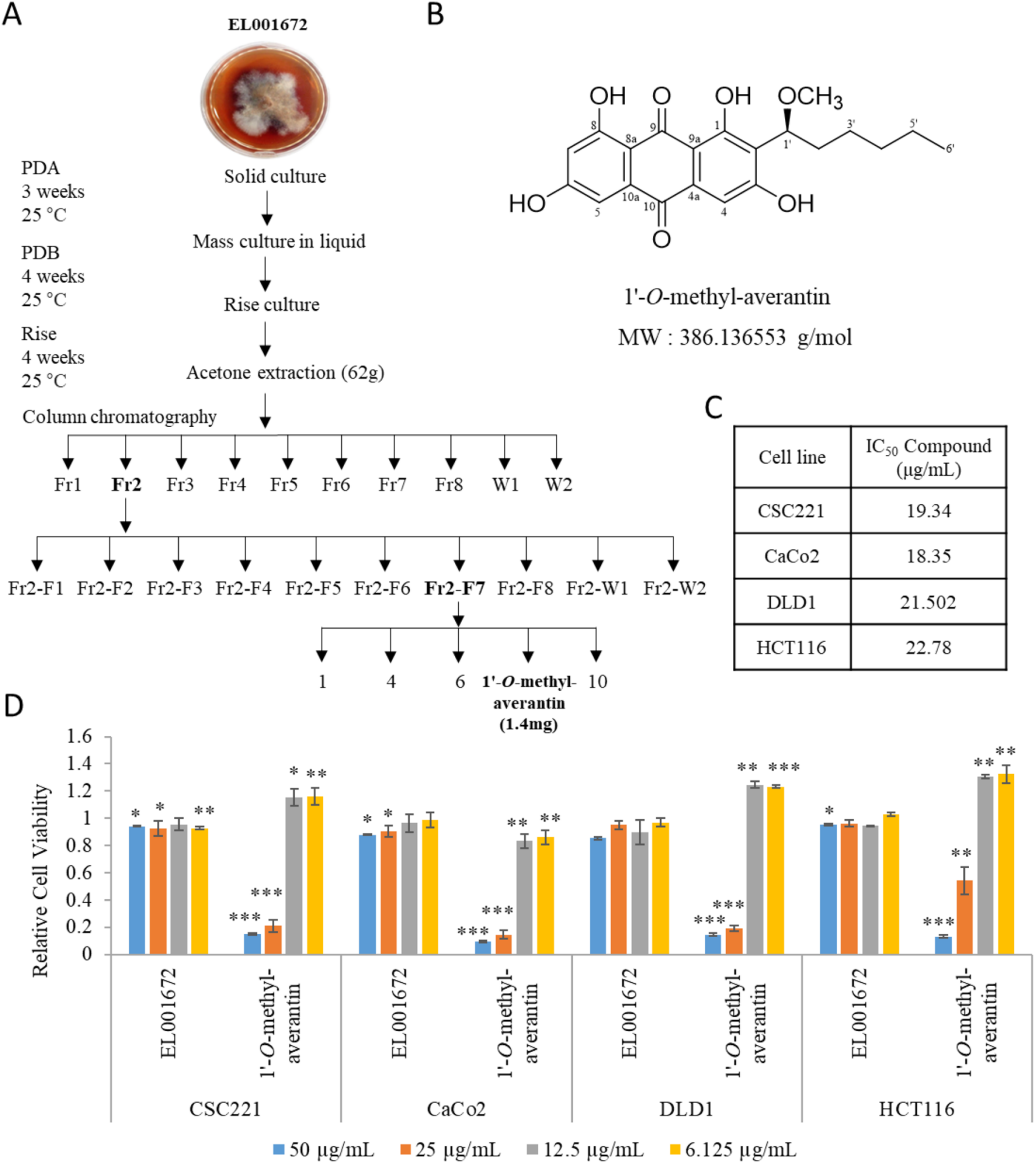
1′-*O*-methyl-averantin, isolated from a crude extract of EL001672, suppresses cancer stemness. (**A**) Images of the endolichenic fungus EL001672 isolated from *Cetraria* sp., and a flow chart illustrating isolation of the pure compound 1′-*O*-methyl-averantin. (**B**) Chemical structure of 1′-*O*-methyl-averantin. (**C**) IC_50_ values of 1′-*O*-methyl-averantin against the CSC221, CaCo2, DLD1, and HCT116 cell lines. (**D**) Viability of cancer cell lines treated with EL001672 crude extract and 1′-*O*-methyl-averantin. DMSO used as a control. 50, 25, 12.5 and 6.125 µg/ml 1′-*O*-methyl averantin represent a concentration of 129.49, 64.74, 32.37 and 16.19 µM, respectively. Data represent the mean ± standard error of the mean, *p < 0.05; **p < 0.01; ***p < 0.001 (compared with DMSO-treated cells).

**Figure 2:**
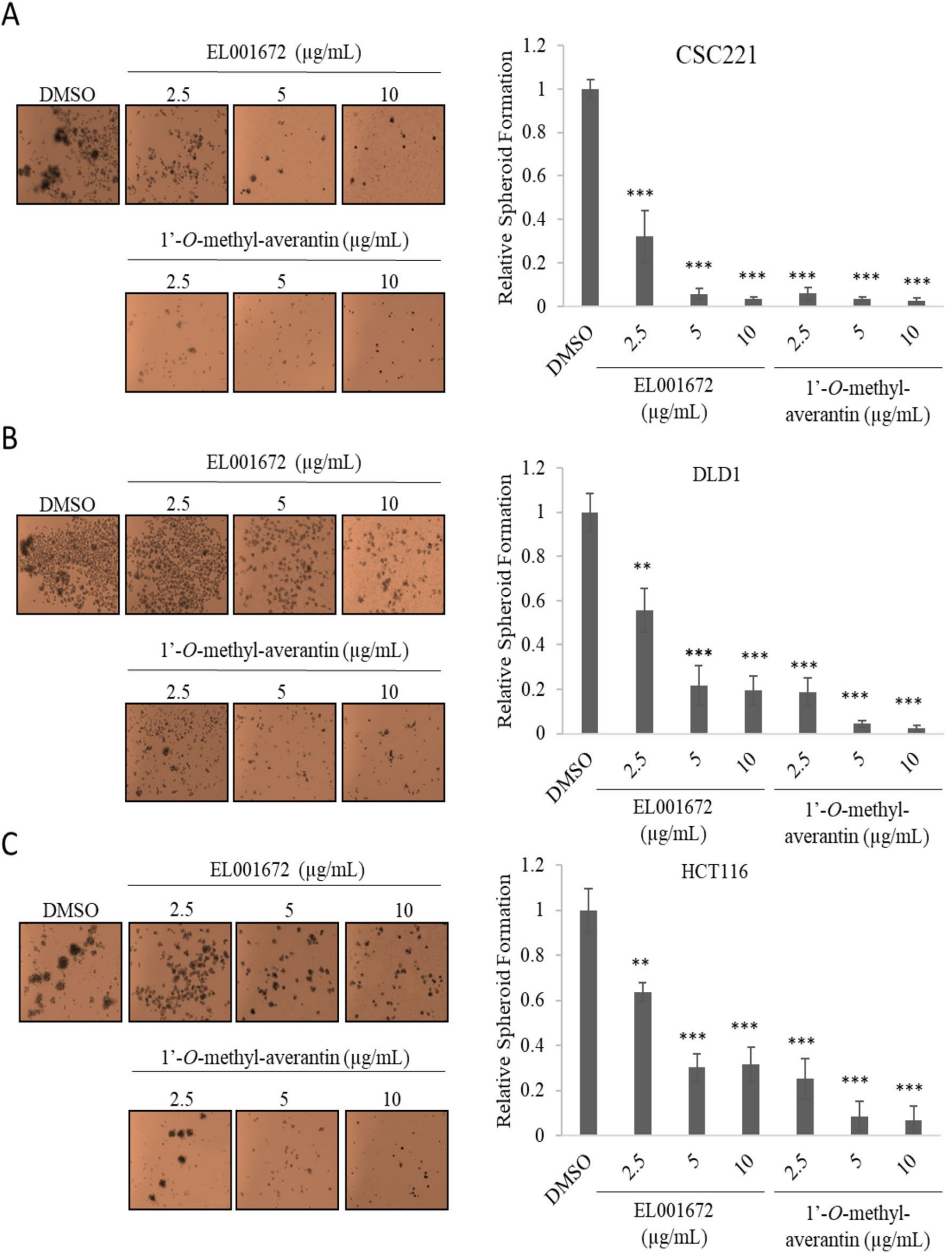
EL001672 extract and 1′-*O*-methyl-averantin inhibit colorectal cancer cell stemness. (**A**–**C**) Representative images of spheroid formation by CSC221, DLD1, and HCT116 cells treated with EL001672 extract or 1′-*O*-methyl-averantin for 14 days, and quantitative analysis of the number of spheroids formed following each treatment. 10, 5 and 2.5 µg/ml 1′-*O*-methyl averantin represent a concentration of 25.90, 12.95 and 6.47 µM, respectively. Data represent the mean ± standard error of the mean, *p < 0.05; **p < 0.01; ***p < 0.001.

**Figure 3:**
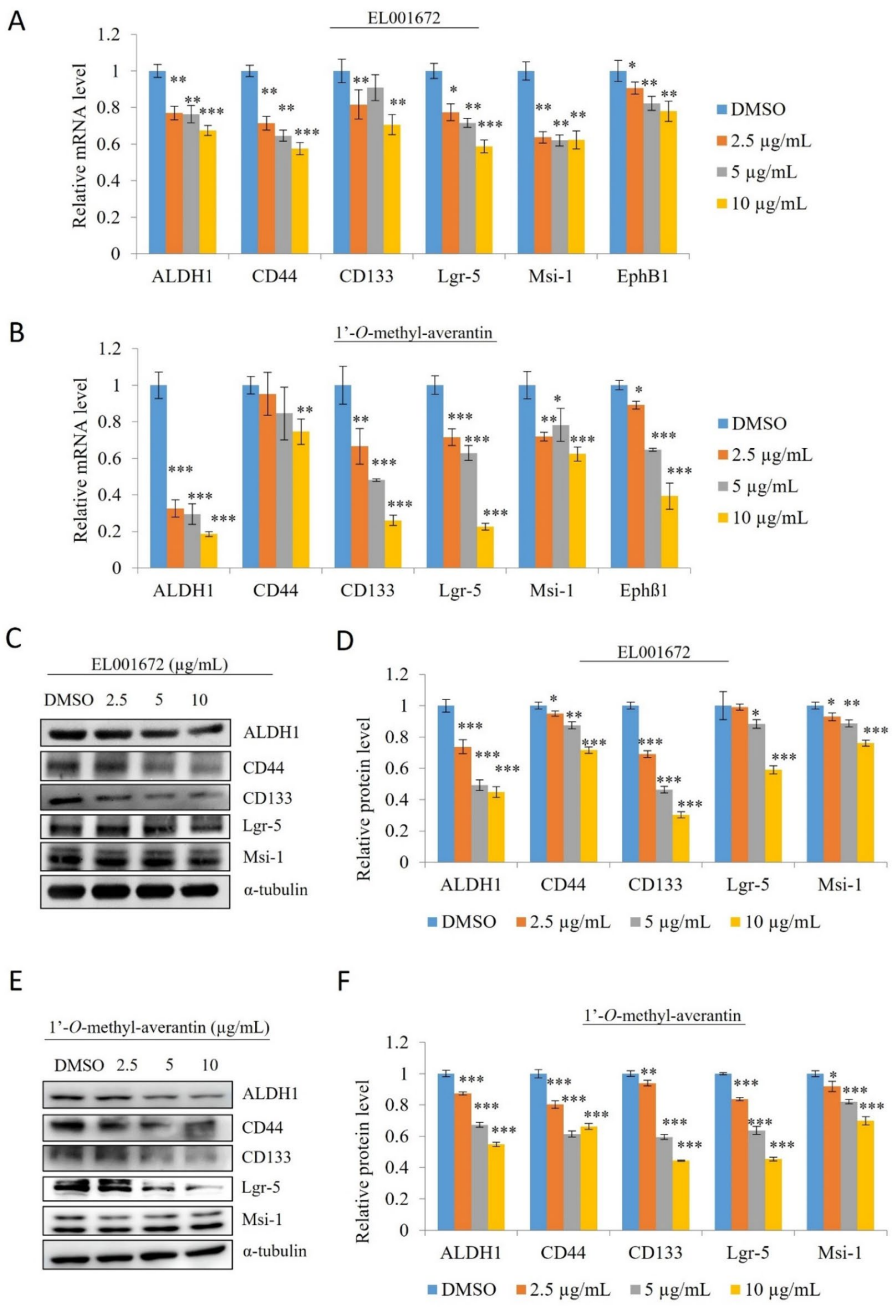
A crude extract of EL001672 and 1′-*O*-methyl-averantin suppress expression of cancer stemness markers. CSC221 cells were treated for 48 h with a crude extract of EL001672 or 1′-*O*-methyl-averantin for mRNA and protein expressions. (**A**,**B**) Quantitative analysis of mRNA encoding cancer stem markers aldehyde dehydrigebase-1 (ALDH1), cluster of differentiation 133 (CD133), CD44, Lgr5, Musashi-1, and EphB1. CSC221 cells were treated for 48 h with a crude extract of EL001672 or 1′-*O*-methyl-averantin. (**C**,**E**) Immunoblots are shown. Relative expression of each target protein after cells were exposed to a crude extract of EL001672 (**D**) or 1′-*O*-methyl-averantin (**F**). 10, 5 and 2.5 µg/ml 1′-*O*-methyl averantin represent a concentration of 25.90, 12.95 and 6.47 µM, respectively. Data represent the mean ± SEM. *p < 0.05; **p < 0.01 (compared with DMSO-treated CSC221 cells).

Bioactivity-guided fractionation of a crude extract from EL001672 led to isolation of compound 1′-*O*-methyl-averantin (Fig. 1A,B, Supple Figs. 1–3). Previous studies suggest that 1′-*O*-methyl-averantin inhibits protein tyrosine phosphates and has weak antioxidant activity^33,35^.

### 1′-***O***-methyl-averantin suppresses cancer stemness

**Figure 4:**
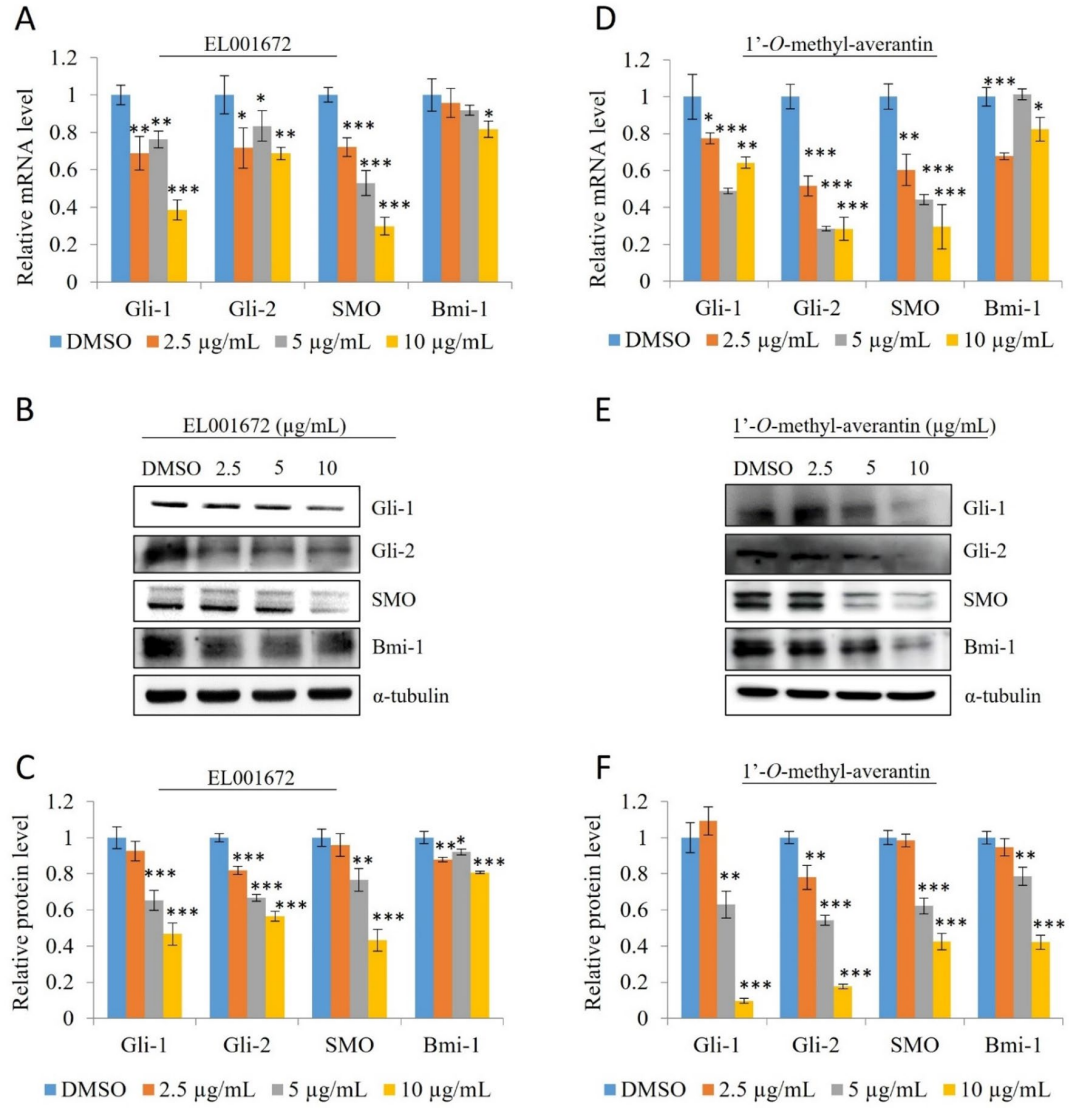
The crude extract of EL001672 and 1′-*O*-methyl-averantin block the sonic Hedgehog (SHH) signaling pathway. (**A**,**D**) Expression of mRNA encoding Gli1, Gli2, SMO, and Bmi-1 by CSC221 cells after treatment with the indicated concentrations of crude extract or 1′-*O*-methyl-averantin and incubated 48 h. (**B**–**E**) Western blot analysis of Gli1, Gli2, SMO, and Bmi-1 protein levels in CSC221 cells treated with indicated concentrations of crude extract and 1′-*O*-methyl-averantin and incubated 48 h. (**C**–**F**) Quantitative analysis of protein expression. 10, 5 and 2.5 µg/ml 1′-*O*-methyl averantin represent a concentration of 25.90, 12.95 and 6.47 µM, respectively. Data represent the mean ± standard error of the mean, *p < 0.05; **p < 0.01; ***p < 0.001; difference compared with DMSO-treated.

MTT assays were performed to investigate the effect of the crude extract and 1′-*O*-methyl-averantin on the viability of colon cancer cell lines CSC221, Caco2, DLD1, and HCT116 for 48 h. The EL001672 crude extract had no significant effect on cell viability; however, 1′-*O*-methyl-averantin exerted cytotoxic effects against all cell lines at 50 μg/mL and 25 μg/mL. The IC_50_ of 1′-*O*-metil-averantin was 19.34 µg/mL in CSC221, 18.35 µg/mL in CaCo2, 21.502 µg/mL in DLD1, and 22.78 µg/mL in HCT116 (Fig. 1C,D). The doubling times of CaCo2, HCT116, and DLD1 cell lines are approximately 62 h, 22 h^36^, 22 h^37^, respectively. Here, 1′-*O*-methyl-averantin has cytotoxic effects in cell lines with different doubling times and may be a potential apoptosis-inducing agent. To further characterize whether 1′-*O*-methyl-averantin has selective cytotoxicity on CRC cells, it needs to test on non-transformed cells. The results tested in HEK293T (human embryonic kidney cells) cells showed that 1′-*O*-methyl-averantin also showed cytotoxic effect at 50 and 25 µg/ml (Supple Fig. 4). These results suggested that further investigations are required to verify the general cytotoxicity of 1′-*O*-methyl-averantin at high concentration. Potential future studies include extensive cytotoxic studies of 1′-*O*-methyl-averantin in different normal and cancer cell lines, as well as research ideas to achieve cancer-specific delivery and/or target-based optimization of the compound to maximize the efficacy and lower the toxicity.

### 1′-***O***-methyl-averantin inhibits spheroid formation in CRC cells

To confirm the concentration-dependent inhibitory effect of 1′-*O*-methyl-averantin and the crude extract on CSC activity in colorectal cancer cell lines, we evaluated spheroid formation by CSC221, DLD1, and HCT116 cells. Both the crude extract and 1′-*O*-methyl-averantin suppressed spheroid formation significantly, suggesting that the EL001672 crude extract and 1′-*O*-methyl-averantin inhibit cancer stemness (Fig. 2A–C).

### 1′-***O***-methyl-averantin suppresses expression of CSC markers

To examine whether the crude extract and 1′-*O*-methyl-averantin inhibit expression of cancer stemness markers in CSC221 cells, we measured expression of mRNA encoding ALDH1, CD133, CD44, Lgr5, Msi-1, and EphB1 (Fig. 3A,B). Both the crude extract and 1′-*O*-methyl-averantin downregulated expression of cancer stemness markers in a dose-dependent manner. However, immunoblot analysis suggested downregulation of ALDH1, CD44, CD133, Lgr-5, and Msi-1 protein expression (Fig. 3C–F).

### 1′-***O***-methyl-averantin inhibits the SHH signaling pathway in CSC221 cells

**Figure 5:**
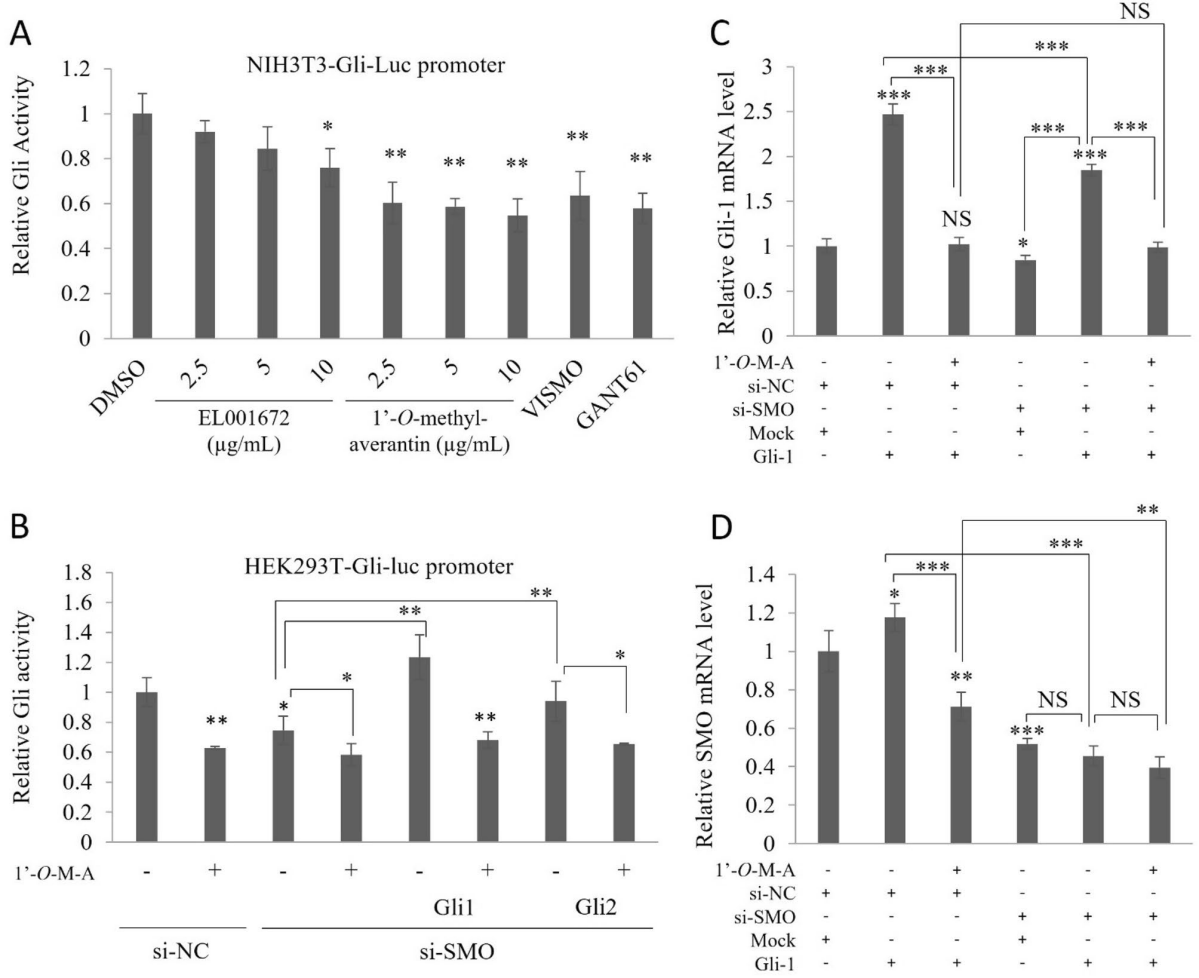
Inhibitory effect of 1′-*O*-methyl-averantin on Gli-overexpression models. (**A**) Gli-luc reporter assays: NIH 3T3 cells stably incorporating Gli-dependent firefly luciferase and constitutive Renilla luciferase reporters were treated with 2.5, 5 or 10 μg/mL crude extract, 1′-*O*-methyl-averantin, 5 μg/mL GANT61 (a Gli inhibitor), and Vismodegib-GDC-0449 (a SMO inhibitor) and incubated 48 h. (**B**) Gli-luc reporter assays in HEK293T. HEK293T cells transfected with si-negative control and/or si-SMO for 12 h, and then cells were exposed to Gli-luc plasmid and Gli1 or Gli2 plasmid transfection for 12 h. After that cell treated with DMSO or 1′-*O*-methyl-averantin for 48 h. (**C**,**D**) Quantitative analysis of mRNA expression levels of Gli1 and SMO on HEK293T cells. HEK293T cells transfected with si-negative control and/or si-SMO for 12 h, and then cells were exposed to Gli1 plasmid and/or mock transfection for 12 h. After that cell treated with DMSO or 1′-*O*-methyl-averantin for 48 h. 10, 5 and 2.5 µg/ml 1′-*O*-methyl averantin represent a concentration of 25.90, 12.95 and 6.47 µM, respectively. Data represent the mean ± standard error of the mean, *p < 0.05; **p < 0.01; ***p < 0.001; difference compared with DMSO-treated.

Next, we examined expression of the SHH pathway genes and proteins, and conducted reporter assays to examine the pathways inhibited by 1′-*O*-methyl-averantin. Changes in the expression of SHH pathways mRNA levels were observed in CSC221 cells treated with the crude extract or 1′-*O*-methyl-averantin. As shown in Fig. 4A and D, expression of Gli1, Gli2, SMO, and Bmi-1 mRNA was downregulated significantly, and in a dose–response manner. Western blot analysis revealed significant downregulation of Gli1, Gli2, SMO, and Bmi-1 protein expression in CSC221 cells (Fig. 4B,C,E,F). Next, we used NIH3T3 cells harboring an integrated Gli reporter gene to examine Gli luciferase activity in the presence of the crude extract, 1′-*O*-methyl-averantin, GANT61 (a Gli inhibitor), and Vismodegib (a SMO inhibitor). 1′-*O*-methyl-averantin suppressed Gli activity significantly at all concentrations tested, with an effect similar to that of the Gli inhibitor (Fig. 5A). Then, we designed Gli-expressed cell models to further characterize how the SHH signaling pathway regulates the upstream flow. 1′-*O*-methyl-averantin suppressed luciferase activity in HEK293T cells, similar to the results of the reporter assay in NIH3T3-Gli-luc cells. Groups of the cell with Gli-luc, Gli1-mediated Gli-luc and Gli-2-mediated Gli-luc were silenced with an SMO-specific si-RNA. Silencing SMO downregulated Gli-luc activity compared to the negative control group, and 1′-*O*-methyl-averantin retained its Gli inhibition ability in the group of cells with si-SMO. Cells overexpressing Gli1 and/or Gli2 restored si-SMO mediated downregulation in Gli-luc activity and compound treatment in this cell successfully downregulated Gli1- and/or Gli2-induced Gli-luc activity. Particularly, 1′-*O*-methyl-averantin more dramatically suppresses Gli1-induced Gli-luc activity (Fig. 5B). For further validation, we checked the levels of Gli1 and SMO mRNA by qRT-PCR in cells overexpressed Gli1 and subsequently in cells in which SMO was silenced. 1′-*O*-methyl-averantin treatment dramatically suppressed Gli1 mRNA levels in Gli1-induced and SMO-silenced or non-silenced cell groups. In addition, there was no statistically significant difference between the inhibition activities of Gli-1 mRNA expression of the SMO-silenced compound treatment group and the non-silenced compound treatment groups. Our results show that inhibition of the SHH signaling pathway downstream effector Gli1 occurs independently of the upstream receptor SMO (Fig. 5C,D). Taken together, these results suggest that the SHH signaling pathway plays a major role in inhibition of colorectal cancer stemness by 1′-*O*-methyl-averantin.

### 1′-***O***-methyl-averantin suppresses the Notch signaling pathway in CSC221 cells

Next, we evaluated the potential inhibitory effect of the crude extract and 1′-*O*-methyl-averantin on the Notch signaling pathway in CSC221 cells. We used quantitative RT-PCR to measure expression of Notch-1 and Hes-1 mRNA. The results showed that expression of both Notch-1 and Hes-1 mRNA was downregulated significantly by the crude extract.

**Figure 6:**
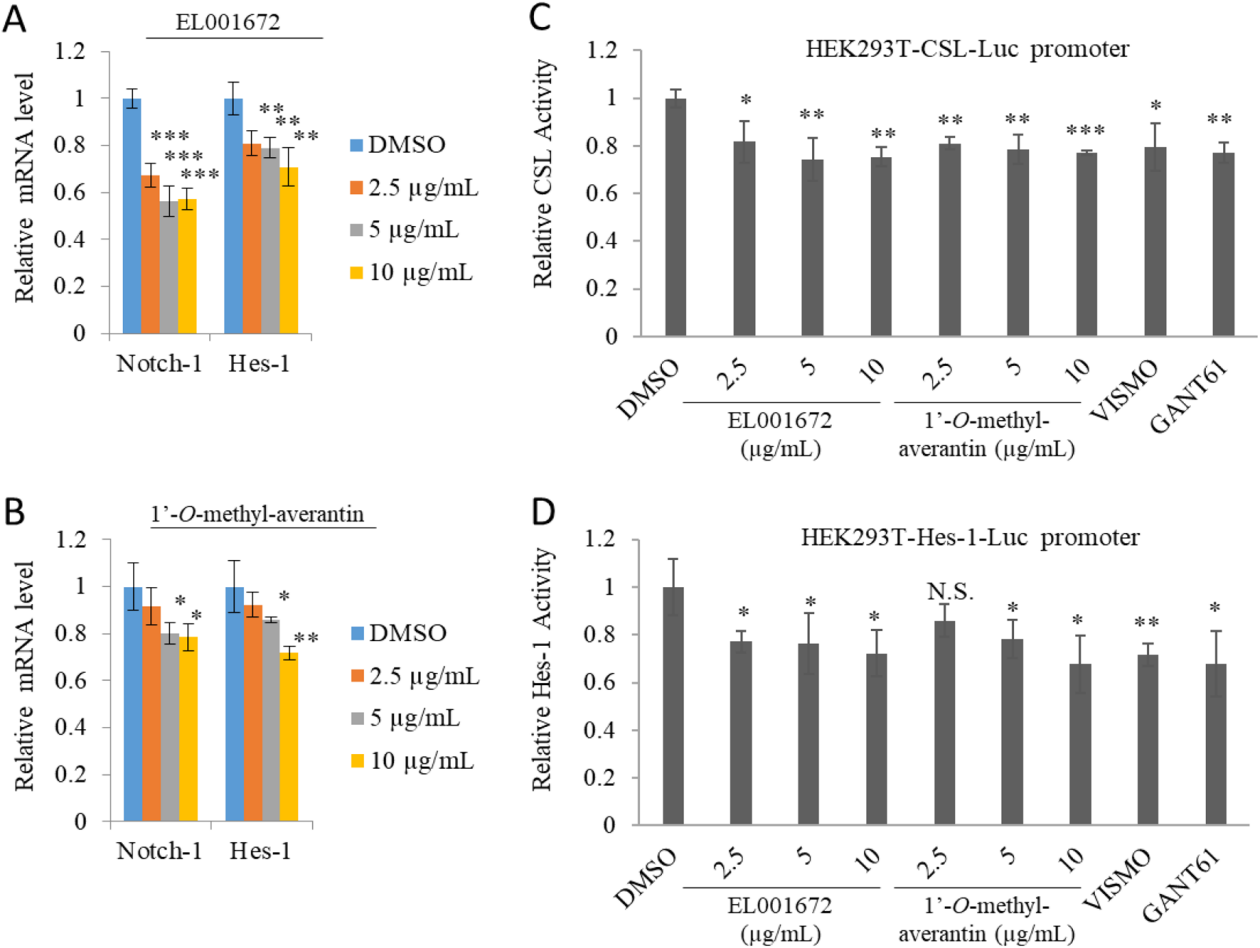
Effect of a crude extract of EL001672 and 1′-*O*-methyl-averantin on the NOTCH signaling pathway. (**A**,**B**) Quantitative analysis of Notch-1 and Hes-1 (hairy and enhancer of split-1) mRNA expression by CSC221 cells treated with the indicated concentrations of crude extract or 1′-*O*-methyl-averantin and incubated 48 h. (**C**,**D**) CSL (CBF1/Su(H)/Lag-1)-luc and Hes-1 reporter assays in HEK293T cells treated with 2.5, 5, or 10 μg/mL crude extract, 1′-*O*-methyl-averantin, 5 μg/mL GANT61 (a Gli inhibitor), and Vismodegib-GDC-0449 (a SMO inhibitor) and incubated 48 h. 10, 5 and 2.5 µg/ml 1′-*O*-methyl averantin represent a concentration of 25.90, 12.95 and 6.47 µM, respectively. Data represent the mean ± standard error of the mean. *p < 0.05; **p < 0.01; ***p < 0.001; difference compared with each groups.

1′-*O*-methyl-averantin tended to downregulate expression of Notch-1 and Hes-1 mRNA at all concentrations tested, but showed statistically significant levels of inhibition at concentrations of 5 and 10 µg/mL (Fig. 6A,B). Next, we used HEK293-transfected cells to conduct a dual luciferase promoter assay to examine suppression of Hes-1 (hairy and enhancer of split-1)-luc and CSL (CBF1/Su(H)/Lag-1)-luc promoter activity (Fig. 6C,D). Crosstalk between the SHH and Notch signaling pathways mean that these pathways can regulate each other^38^. Therefore, we compared the inhibitory activity of Gli and SMO inhibitors with that of the crude extract and 1′-*O*-methyl-averantin. The crude extract and 1′-*O*-methyl-averantin significantly downregulated Hes-1-luc and CSL-luc promoter activity to levels observed for the Gli and SMO inhibitors.

## Discussion

Many compounds of lichen origin have effects on tumor progression, cell cycle progression, apoptosis, angiogenesis, energy metabolism, and immune modulation^39–42^. In this study, we isolated 1′-*O*-methyl-averantin from an endolichenic fungi acetonic extract of *Cetraria* sp. Previous studies show that an unidentified marine-derived fungus ZSUH-36 isolated from *Acanthus ilicifolius Linn*., and the marine-derived fungus *Aspergillus* sp. SCSIO F063, produce 1′-*O*-methyl-averantin^34,43^. 1′-*O*-methyl-averantin has structural similarities with many compounds and is classified as an anthraquinone^44^. Anthraquinone derivatives have antitumor activity and cytotoxic activity, as well as effects on the cell cycle and apoptosis^45–49^. However, no study has examined the anticancer activity of 1′-*O*-methyl-averantin in detail. Here, we evaluated the effect of a crude extract of EL001672, as well as 1′-*O*-methyl-averantin, on colorectal cancer cells. Several colorectal cancer cell lines were used in this study. DLD1 has mutant KRAS, BRAF, PIK3CA, TP53, and APC and MSI*;* Caco2 has wild-type KRAS, BRAF, PIK3CA, TP53, APC and MSS; HCT116 has KRAS mutant; CSC221 is human colorectal adenocarcinoma-enriched cancer stem cell^50,51^. The results were as follows: (1) 1′-*O*-methyl-averantin was cytotoxic to CSC221, CaCo2, DLD1, and HCT116 cells; (2) 1′-*O*-methyl-averantin and the crude extract suppressed spheroid formation by colorectal cancer cells; (3) 1′-*O*-methyl-averantin and the crude extract downregulated expression of cancer stem cell markers; (4) 1′-*O*-methyl-averantin and the crude extract inhibited the SHH signaling pathway was markedly inhibited with 1′-*O*-methyl-averantin in CSCs cell; and (5) 1′-*O*-methyl-averantin and the crude extract inhibited Notch signaling at the transcriptional level. Collectively, the data show that 1′-*O*-methyl-averantin suppresses colorectal cancer stemness by regulating the SHH and Notch signaling pathways, suggesting that it may have potential as a therapeutic drug.

CSCs drive cancer formation and metastasis. Therefore, developing treatment modalities that target CSCs has great potential^52,53^. The SHH and Notch signaling pathways play major roles in cancer development, and so are themselves a possible therapeutic target^54,55^. The transmembrane response to the SHH ligand is modulated and mediated by two transmembrane proteins: PTCH and SMO. Gli1 and Gli2 are downstream targets of SHH signaling^56^. In this study, we measured expression of SMO, Gli1, and Gli2 mRNA and protein, and found that both were inhibited by 1′-*O*-methyl-averantin and the crude extract. Moreover, 1′-*O*-methyl-averantin and crude extract inhibited Gli reporter activity in stable Gli-overexpressing cells. The inhibitory activity of 1′-*O*-methyl-averantin at 2.5, 5, and 10 μg/mL was similar to that of a Gli inhibitor (GANT61) and an SMO inhibitor (Vismodegib) used at 5 μg/mL. On the other hand, the crude extract can significantly suppress Gli reporter activity at a concentration of 10 μg/ml, but has a weaker inhibitory effect than 1′-*O*-methyl-averantin. The anti-stemness activity of 1′-*O*-methyl-averantin was suppressed the SHH pathway, with SMO, Gli1, and Gli2. Moreover, 1′-*O*-methyl-averantin targets the SHH signaling pathway via Gli1, independently of SMO. SHH signaling increases Bmi-1 expression, which in turn drives cellular reprogramming^57,58^. In particular, the downstream transcription factor Gli within the SHH pathway induces expression of Bmi-1^59–61^. Our results suggest that after treatment 1′-*O*-methyl-averantin and crude extract, Bmi-1 expression tended to be downregulated.

Studies reported that crosstalk between the SHH and Notch signaling pathways results in them regulating each other^62–64^. Therefore, we examined the effects of the pure compound and crude extract on Notch signaling. When notch ligands bind to Notch receptors, γ-secretase liberates the intracellular domains of the NICD (Notch receptor) via proteolytic cleavage (S3 cleavage). The NICD undergoes nuclear translocation and associates with the transcription factor CSL. The NICD/CSL complex activates Hes1 and other Notch signaling target genes^65–67^. Here, we used qRT-PCR and a reporter assay to evaluate inhibition of Notch signaling by 1′-*O*-methyl-averantin and the crude extract. The results showed that Notch-1 and Hes-1 mRNA tended to be downregulated after treatment. In addition, CSL and Hes-1 reporter activity were inhibited by both the crude extract and 1′-*O*-methyl-averantin.

ALDH1, CD133, CD44, Lgr5, Msi-1, and EphB1 are markers of cancer stemness^3,8,68,69^. Human colon tumors are characterized by CD133+CD44+ cells, which are known as CSCs or tumor-starting cells^70,71^. The G-protein-coupled receptor family of proteins includes the leucine-rich repeat-containing G protein-coupled receptor 5 (Lgr5), also known as GPR49. Lgr5 is a target of Wnt signaling, and a selective and ideal cancer stem cell marker^72,73^. EphB receptors coordinate proliferation and migration within the intestinal stem cell niche^74–76^. Our data suggest that 1′-*O*-methyl-averantin downregulates expression of multiple stem cell markers associated with colorectal cancer.

ALDH1 levels are associated with liver metastases, tumor progression, drug resistance, and a poor clinical outcome^77,78^. Studies suggest that deletion of Gli-1 reduces expression of ALDH1 and CD44^79^, and that ALDH1 can modulate expression of Gli^80^. The novel inhibitors GANT61 (inhibitor of Gli1 and Gli2 targeting the Hedgehog/GLI pathway) and DAPT (inhibitor of Notch, a γ-secretase substrate) downregulate ALDH1 expression^81,82^. Our results show that 1′-*O*-methyl-averantin significantly downregulated expression of ALDH1 at both the mRNA and protein levels. Previous studies tried to identify the mechanism underlying the links between ALDH1-SHH and ALDH1-Notch^82–88^; the data presented in the present study suggest that 1′-*O*-methyl-averantin and the crude extract have co-inhibitory effects on ALDH1, SHH, and Notch signaling.

## Materials and methods

### General experimental procedures

NMR spectra were acquired by containing Me_4_Si as internal standard on Varian Inova spectrometers 400 MHz and 100 MHz spectrometer (Varian Medical Systems, Inc., Virginia, USA) using solvent DMSO-*d*_*6*_ (Cambridge Isotope Laboratories (CIL), Inc., Tewksbury, MA, USA). Low-resolution LC–MS measurements were performed using the Agilent Technology 1260 quadrupole (Agilent Technologies, Santa Clara, CA, USA) and Waters Alliance Micromass ZQ LC–MS system (Waters Corp, Milford, MA, USA) using reversed-phase column (Phenomenex Luna C18 (2) 100 Å, 50 mm × 4.6 mm, 5 µm) (Phenomenex, Torrance, CA, USA) at a flow rate 1.0 mL/min at the National Research Facilities and Equipment Center (NanoBioEnergy Materials Center) at Ewha Womans University. Optical rotations were acquired using a Kruss Optronic P-8000 polarimeter with a 5-cm cell (Kruss, Habrug, Germany).

The compound is an orange-red amorphous powder. LR-ESI–MS spectroscopic data revealed an ionic peak at *m/z* 372.3 [M+H]^+^. The ^1^H NMR spectrum of 1′-*O*-methyl-averantin displayed two meta-coupled aromatic protons [*δ*_H_ 7.10 (1H, d, *J* = 2.3 Hz, H-5), three hydroxyl groups [*δ*_H_ 12.82 (1H, s, 1-OH), *δ*_H_ 12.15 (1H, s, 8-OH), and *δ*_H_ 6.58 (1H, d, *J* = 2.3 Hz, H-7)], and one methoxy proton [*δ*_H_ 3.15 (3H, s, 1′-OCH_3_)]. The ^13^C NMR spectrum of 1′-*O*-methyl-averantin revealed 12 carbons. HSQC spectra revealed two carbonyl carbons [*δ*_C_ 188.8 (C-9), and *δ*_C_ 181.4 (C-10)], a methoxy carbon [*δ*_C_ 56.3 (C-OCH_3_)], nine fully-substituted carbons [*δ*_C_ 165.4 (C-8), *δ*_C_ 164.4 (C-1), *δ*_C_ 164.0 (C-6), *δ*_C_ 163.2 (C-3), *δ*_C_ 133.5 (C-10a), *δ*_C_ 134.9 (C-4a), *δ*_C_ 119.4 (C-2), *δ*_C_ 108.8 (C-8a), and *δ*_C_ 108.1 (C-9a)], four methylene carbons [*δ*_C_ 32.5 (C-2′), *δ*_C_ 31.2 (C-4′), *δ*_C_ 25.4 (C-3′), and *δ*_C_ 22.1 (C-5′)], four methine carbons [*δ*_C_ 108.5 (C-5), *δ*_C_ 108.7 (C-4), *δ*_C_ 108.2 (C-7), and *δ*_C_ 74.6 (C-1′)], and one methyl carbon [*δ*_C_ 13.9 (C-6′)]. Based on a comparison with NMR data in the literature, the compound was identified as 1′-*O*-methyl-averantin^89,90^. The NMR datas are available in Supplementary Figs. 5–9. Optical rotation of **1** (${[\mathrm{\alpha }]}_{\mathrm{D}}^{21}$ = + 24.09°, c 0.11, CH_3_OH) suggested that the absolute configuration at C-1′ should be an (*R*)-configuration^90^**.**

### Fungal strain

Lichen specimen of *Cetraria* sp. was collected from the Yunnan, baima snow mountain. Voucher specimens were deposited in the Korean Lichen Research Institute, Suchon National University, Korea (https://cc.aris.re.kr/kolabic). The endolichenic fungus EL001672 was isolated from the lichen specimen of *Cetraria* sp. using the surface sterilization method^91^.

As already shown, fungus internal transcribed spacer sequencing was carried out^25^. DNA was extracted from EL0001672 cultured on PDA using a DNeasy Plant Mini Kit following the manufacturer's protocols (Qiagen, Hilden, Germany). The internal transcribed spacer (ITS) region of the rDNA was amplified with the universal primers ITS1F (5′-CTTGGTCATTTAGAGGAAGTAA-3′)^92^ and LR5 (5′-ATCCTGAGGGAAACTTC-3′)^93^. EL001672, was determined as *Jackrogersella* sp. by the BLAST search of the ITS sequence, which showed 98.99% similarity to that of *Jackrogersella multiformis* (GenBank Accession No. ON453667.1). The ITS sequence of *Jackrogersella* sp. EL001672 is available in Supplementary Table 1.

### Preparation of endolichenic fungal extracts

Firstly, EL001672 grew on potato dextrose agar (PDA) medium at 25 °C. Mycelial growth was monitored in 500 ml Erlenmeyer flasks containing 200 ml of potato dextrose broth (PDB) incubated at 25 °C on a rotary shaker at 150 rpm for 4 weeks. Next, EL001672 cultured in liquid culture was subjected to rice culture for 4 weeks. Separation of mycelium on the surface was achieved. The mycelia were dried and then extracted with acetone. To produce the crude extract (62 g), the organic phase was then evaporated to dryness under a vacuum. For use in additional investigations, the dried extracts were then completely dissolved in DMSO.

### Isolation of active compound from EL001672

The crude extract of EL001672 (62 g) was fractionated by the flash column chromatography on silica gel eluted with CH_2_Cl_2_/CH_3_OH (100/0, 100/2, 100/1, 100/5, 100/10, 100/20, 100/50, 100/100 and 0/100, v/v, each of 600 mL) to obtain eight fractions (Fraction 1 to Fraction 8). The fraction two (7.88 g) was further fractionated by the flash column chromatography on C-18 resin eluted with H_2_O/CH_3_OH (80/20, 60/40, 50/50, 40/60, 30/70, 20/80, 0/100, each of 600 mL) to obtain eight subfractions (Fraction 1 to Fraction 8). The subfraction seven was isolated by reversed-phase HPLC (Phenomenex Luna C-18 (2), 250 × 100 mm, 2.0 mL/min, 5 μm, 100 Å, UV = 280 nm) using an isocratic condition 85% CH_3_CN in H_2_O to obtain 1′-*O*-methyl-averantin (1.4 mg, t_*R*_ = 22.5 min).

### Cell culture and Reagents

CSC221 (human colorectal adenocarcinoma-enriched CSCs), CaCo2 (MSS and wild-type KRAS, BRAF, and PIK3CA), DLD1 (MSI and mutant KRAS and PIK3CA), HT116 (KRAS mutation), HEK293T (human embryonic kidney cells) cells were used in this research. CSC221 cell was cultured in DMEM (GenDepot, Katy, TX, USA) supplemented with 10% fetal bovine serum (FBS) and 1% penicillin streptomycin solution. HEK293T, Caco2, DLD1, and HCT116 cell lines were purchased from the American Type Culture Collection (ATCC, Manassas, VA, USA). CSC221 cell line was purchased from BioMedicure (San Diego, CA, USA). Cells were incubated in 5% CO2 in a humidified atmosphere at 37 °C^51^. GANT61 (G9048) was purchased from Sigma-Aldrich and Vismodegib-GDC-0449 (A10258) was purchased from AdooQ Bioscience (Irvine, CA, USA).

### MTT assay

Cells were seeded at a density of 2.5 × 10^3^ cells/well in 96-well plates, after overnight cells were treated with crude extract of EL001672 and 1′-*O*-methyl-averantin for 48 h incubation. The 3-(4,5-dimethylthiazol-2-yl)-2,5-diphenyltetrazolium bromide (MTT) was added and maintained for 4 h once treatment was completed. DMSO was added after removing the medium. Absorbance at 570 nm was determined using a microplate reader with Gen 5 (2.03.1) software (BioTek, Winooski, VT, USA)^94,95^.

### Spheroid assay

Cells were trypsinized at that point washed with N2-supplemented DMEM/F12 (Invitrogen, Carlsbad, CA, USA). Human basic fibroblast growth factor (hbFGF; 10 ng/mL; Invitrogen) and human recombinant epidermal growth factor (hrEGF; 20 ng/mL; Biovision, Atlanta, GA, USA) are added in N2-supplemented DMEM/F12^96^. After CSC221 cells seeded at a density of 5 × 10^3^ cells/well in ultra-low attachment 24-well plates (Corning Inc., Corning, NY, USA). After 14 days of incubation, spheres were quantitated by inverted phase contrast microscopy. The relative sphere formation ability was calculated through the IMT iSolution software (IMT iSolution Inc., Northampton NJ, USA)^97^ measuring the pixel intensity of the sphere area randomly in each plate. Data are presented as the average of three independent experiments.

### Western blotting

CSC221, cells treated for 48 h were washed twice with ice-cold phosphate-buffered saline (PBS) and lysed in lysis buffer^24^. Antibodies against ALDH1 (sc-166362; Santa Cruz Biotechnology, Dallas, TX, USA), CD133 (CA1217; Cell Applications, San Diego, CA, USA), CD44 (3570; Cell Signaling Technology, Danvers, MA, USA), Lgr-5 (ab75850; Abcam, Cambridge, MA, USA), Msi-1 (ab52865, Abcam), Gli1 (sc-20687; SANTA CRUZ, Dallas, TX, USA), Gli2 (sc-271786; SANTA CRUZ), Smoothened (SMO; ab72130; Abcam, Cambridge, MA, USA), Bmi-1 (ab38295; Abcam) were used to detection. α-tubulin (2125, Cell Signaling Technology) and β-Actin (sc-47778; SANTA CRUZ) antibody was used as an internal control. The bands were cut according to the protein size region of interest before intubating with antibodies and then imaged with an Immobilon Western Chemiluminescent HRP Substrate Kit (Merck Millipore, Billerica, MA, USA). Uncropped images of the blot images are presented as additional data (Supple Figs. 10, 11). Bands relative density was calculated based on the density of α-tubulin and actin bands in each sample. Values were demonstrated as arbitrary densitometric units corresponding to signal intensity.

### Plasmids, si-RNA and reporter assay

SMO siRNA (human specific) and si-negative control purchased from Santa Cruz Biotechnology. The si-RNA and si-negative control were transfected by Lipofectamine RNAiMAX protocol. Gli1 and Gli2 plasmid was transfected into HEK293T cells using X-treme GENE 9 DNA.

NIH3T3-Gli-luc cells (stably incorporating Gli-dependent firefly luciferase and constitutive Renilla luciferase reporters) using for Gli reporter activity. HEK293T cells were transfected with Hes-1-, and CSL-conjugated firefly plasmid along with *Renilla*-luc (pRL-TK) plasmid using the X-treme GENE 9 DNA transfection reagent (Roche, Werk Penzberg, Germany). At 24 h after transfection, cells were treated with crude extract of EL001672 and 1′-*O*-methyl-averantin, or DMSO and incubated for 48 h at 37 °C under 5% CO_2_. Normalized luciferase activity was obtained against Renilla activity to determine the transfection efficiency using a Dual-Luciferase reporter assay system (Promega, Madison, WI, USA)^98^.

### Quantitative reverse-transcription PCR (qRT-PCR)

Quantitative RT-PCR (qRT-PCR) was conducted as already depicted^3^. Total RNA was isolated from CSC221 cells using RNAiso Plus (TaKaRa, Otsu, Japan). 1 mg of RNA was converted to cDNA using M-MLV reverse transcriptase (Invitrogen, Carlsbad, CA, USA). qPCR was performed using SYBR Green (Enzynomics, Seoul, Korea)^99^. Primers for real-time PCR are listed in Supplementary Table 2. qRT-PCR reactions and analyses were performed on a CFX instrument (Bio-Rad, Hercules, CA, USA)^100^.

### Statistical analysis

All experiments were performed in triplicate. Data are expressed as means ± standard deviation. All statistical analyses were performed using the Sigma Plot software^101^.

## Conclusion

In this study, 1′-O-methyl-averantin isolated from *Jackrogersella* sp. EL001672 by followed bioactivity-guided fractionation method. *Jackrogersella* sp. EL001672 and 1′-*O*-methyl-averantin suppresses the cancer stemness markers such as ALDH1, CD44, CD133, Lgr-5, Msi-1 and EphB1. In particular, we focused on the SHH signaling pathway that regulates cancer stem cells. Our results showed that crude extract and 1′-*O*-methyl-averantin had inhibitory activity on SHH signaling pathway regulators Gli1, Gli2 and SMO. Furthermore, we identified that 1′-*O*-methyl-averantin regulates the SHH signaling pathway by targeting the Gli1 downstream effector independently of SMO. Finally, we demonstrated the inhibition of the crude extract and 1′-*O*-methyl-averantin on the upstream receptor NOTCH and the downstream effector Hes-1 and the CSL complex. Taken together, the data suggest that 1′-*O*-methyl-averantin isolated from endolichenic fungi suppresses development of CSC by inhibiting the SHH signaling pathway, making it a candidate novel therapeutic agent.

## Supplementary Information

Supplementary Information.

## Data Availability

All data generated or analysed during this study are included in this published article. All authors read and approved the final manuscript.

## Abbreviations

ALDH1: Aldehyde dehydrogenase 1 family
CSC: Cancer stem cell
CD133: Cluster of differentiation 133
DMSO: Dimethyl sulfoxide
EPHB1: Ephrin type-B receptor 1
Lgr-5: G-protein coupled receptor-5
SMO: G-protein–coupled receptor smoothened
Gli: Gli family zinc finger
HES: Hairy and enhancer of split
ITS: Internal transcribed spacer
NMR: Nuclear magnetic resonance
PTCH1: Protein patched homolog 1
PDA: Potato dextrose agar
PDB: Potato dextrose broth
SHH: Sonic Hedgehog
