# Supplementary material for: 1′-O-methyl-averantin isolated from the endolichenic fungus Jackrogersella sp. EL001672 suppresses colorectal cancer stemness via sonic Hedgehog and Notch signaling
Source: Sci Rep. 2023 Feb 16;13:2811. doi: 10.1038/s41598-023-28773-z (PMC9935543; doi:10.1038/s41598-023-28773-z)
Supplement: Supplementary file 1 — Supplementary Information. [file 41598_2023_28773_MOESM1_ESM.pdf]

Supplementary Materials

1'-*O*-methyl-averantin isolated from the endolichenic fungus *Jackrogersella* sp. EL001672 suppresses colorectal cancer stemness via sonic hedgehog and Notch signaling

Mücahit Varlı[1], Eun-Young Lee[2], Yi Yang[1], Rui Zhou[1], İsa Taş[1], Sultan Pulat[1], Chathurika D. B. Gamage[1], So-Yeon Park[1], Jae-Seoun Hur[3], Sang-Jip Nam[2, *], Hangun Kim[1,*]

[1]College of Pharmacy, Sunchon National University, 255 Jungang-ro, Sunchon, Jeonnam 57922, Korea; mucahitvarli@s.scnu.ac.kr (M.V.); yangyi_520@hotmail.com (Y.Y); zhourui274@gmail.com (R.Z.); mr.isatas@gmail.com (İ.T.); sultanpulat@s.scnu.ac.kr (S.P); chathurika.gamage@gmail.com (C.D.B.G.); sinbu17@naver.com (S.-Y.P.)

[2]Department of Chemistry and Nanoscience, Ewha Womans University, Seoul 03760, Republic of Korea; younglee0124@naver.com (E.-Y.L.); sjnam@ewha.ac.kr (S.-J.N.)

[3]Korean Lichen Res. Institute, Sunchon National University, 255 Jungang-ro, Sunchon, Jeonnam 57922, Korea; jshur1@sunchon.ac.kr (J.S.H.)

*Correspondences: sjnam@ewha.ac.kr (S.-J.N.); Tel.: +82-2-3277-6805;

hangunkim@sunchon.ac.kr (H.K.); Tel.: +82-61-750-3761

Supplementary Materials:

Supplementary Table 1.

ITS sequence of *Jackrogersella multiformis* EL001672

Supplementary Table 2.

Primer list for q-RT-PCR

Supplementary Figure 1-3.

Bioactivity-guided fractionation of a crude extract from EL001672

Supplementary Figure 4.

Viability of HEK293T cells treated with 1'-*O*-methyl-averantin

Supplementary Figure 5-9.

The NMR datas of 1'-*O*-methyl-averantin

Supplementary Figure 10-11.

Full-length blots shown in Figure 3 and Figure 4.

Supplementary Table 1. ITS sequence of *Jackrogersella multiformis* EL001672

Internal transcribed spacer sequence
TCAAAAGGAACCAAGAATTTTTTTTGATTTTTTATTTTGTTCTTACTTTTTTGTT GGTGGGGCCTTGTTTTTCTTTTTTTTTTTGTTTCCTTTCCCGGTTTTGATATGCTT AAGTTCAGCGGGTATTCCTACCTAATCCGAGGTCAACCACTAGAAATATAGGG GGTTTTACGGCAGGCAGCTAAGGCGACTGCAGAAGCGAGAAAAGATTTACTAC GCTCAGAGTACGCCATAACTCCGCCACTGACTTTGAGGAGCTACGCTATAACC GTAGGCTCCCAACGCTAAGCTACAGGGCTTAATGGTCGAAATGACGCTCGAAT AGGCATGCCCACTAGAATACTAATGGGCGCAATGTGCGTTCAAAGATTCGATG ATTCACTGAATTCTGCAATTCACATTACTTATCGCATTTCGCTGCGTTCTTCATC GATGCCAGAACCAAGAGATCCGTTGTTGAAAGTTTTAACTTATTTTTGTTTGGA GCATTCAGAAATACACTATTTTAAACAGAGTTTAGGGGTCCTCCGGCGGGCCG TGAGCCGGCTACAGGGTAGCCGCCCGTTTTACAGGGCGGGCTACAGGGTAGCC GCAGCGAGCGCCGAGGCAACTGGAGGTAAGGTTCACATAGGGTTTGGAGTTTA GTTTAACTCTGTAATGATCCCTCCGCTGGTTCACCAACGGAGACCTTGTTACGA CTTTTACTTCCTCTAGGAAACCCAAAAAATTGTGGGGGTTAAAAGGAAGGCGA CGCCTACACC

Supplementary Table 2. Primer list for q-RT-PCR

Gene Symbol	Primers sequences	
	For (5'-3')	Rev (5'-3')
GAPDH	ATCACCATCTTCCAGGAGCGA	AGTTGTCATGGATGACCTTGGC
ALDH1	TGTTAGCTCATGCCGACTTG	TTCTTAGCCCGCTCAACACT
CD44	TGCCGCTTTGCAGGTGTAT	GGCCTCCGTCCGAGAGA
CD133	GGACCCATTGGCATTCTC	CAGGACACAGCATAGAATAATC
Lgr-5	CTCTTCCTCAAACCGTCTGC	GATCGGAGGCTAAGCAACTG
Msi-1	ACCAAGAGATCCAGGGGTTT	TCGTTCGAGTCACCATCTTG
Gli-1	CCATACATGTGTGAGCACGA	GGCACAGTCAGTCTGCTTT
Gli-2	CAACGCCTACTCTCCCAGAC	GAGCCTTGATGTACTGTACCAC
SMO	CATCCCTGACTGTGAGATCA	CACCATCTTGGTGACATGCT
Hes-1	CTGAAGAAAGAT AGCTCGCG	ACTTCCCCAGCACACTT
Bmi-1	CCA GGGCTTTTCAAAAATGA	CCGATCCAATCTGTTCTGGT
EphB-1	TGCAAGGAGACCTTCAACCT	CGGTGTTGATTTTCATGACG
Notch-1	TCAGCGGGATCCACTGTGAG	ACACAGGCAGGTGAACGAGTTG

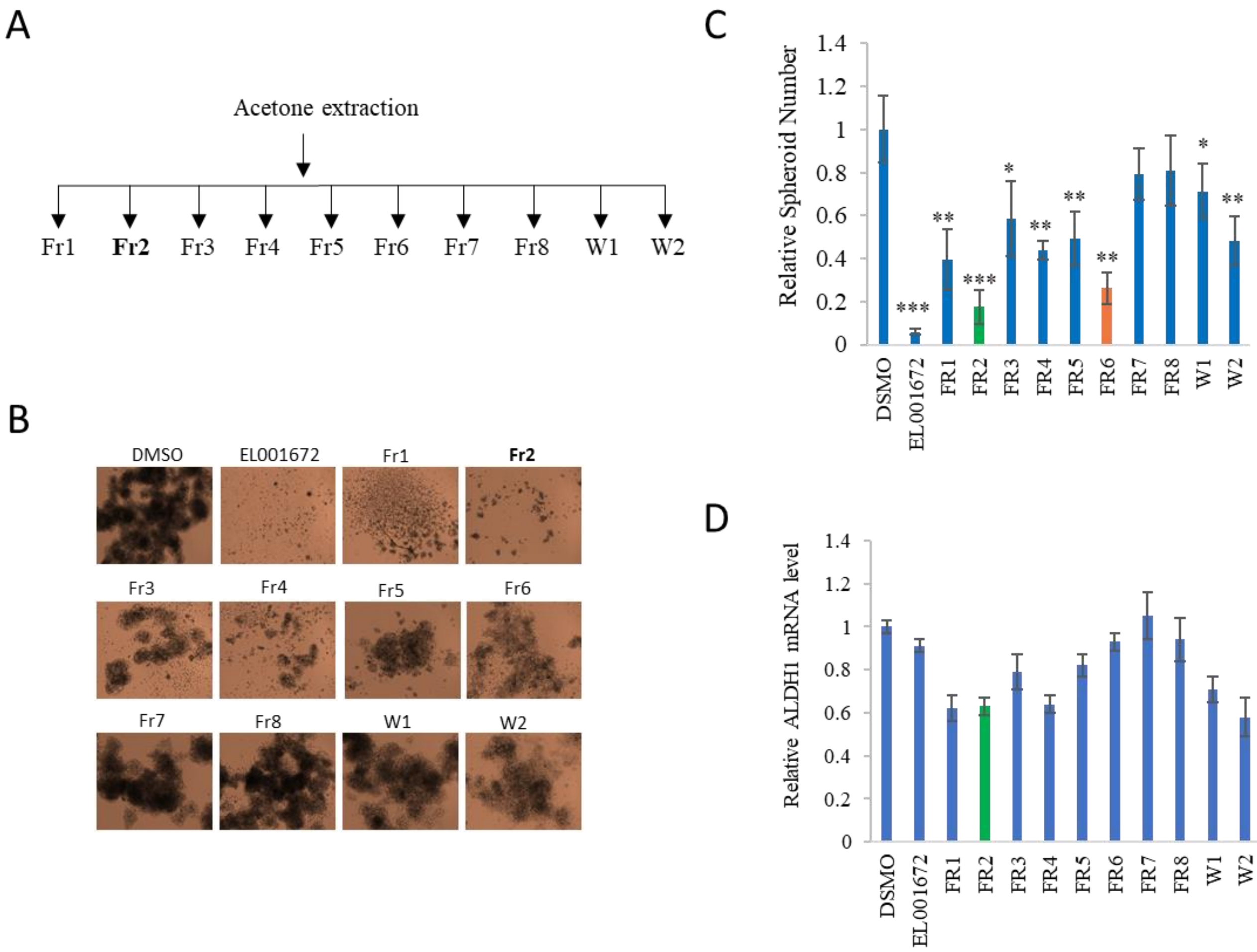

Supplementary Figure 1. (A) Flow chart of the fractionation. (B-C) Representative images of spheroid formation of CSC221 cells treated with fractions for 14 days and quantitative analysis of the number of spheroids following each treatment. (D) Quantitative analysis of mRNA expression levels of cancer stem markers aldehyde dehydrogenase-1 (ALDH1).

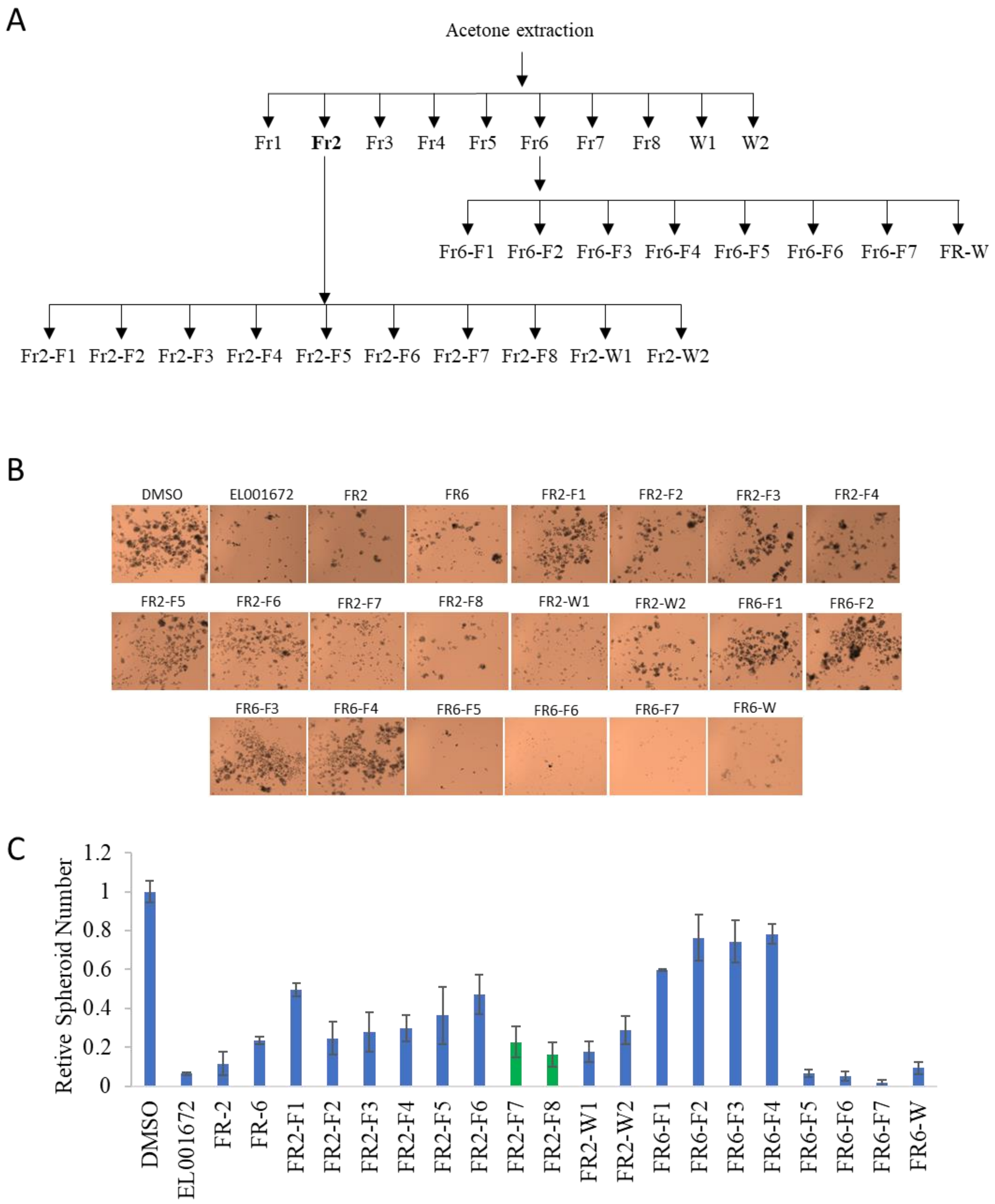

Supplementary Figure 2. (A) Flow chart of the fractionation. (B-C) Representative images of spheroid formation of CSC221 cells treated with fractions for 14 days and quantitative analysis of the number of spheroids following each treatment.

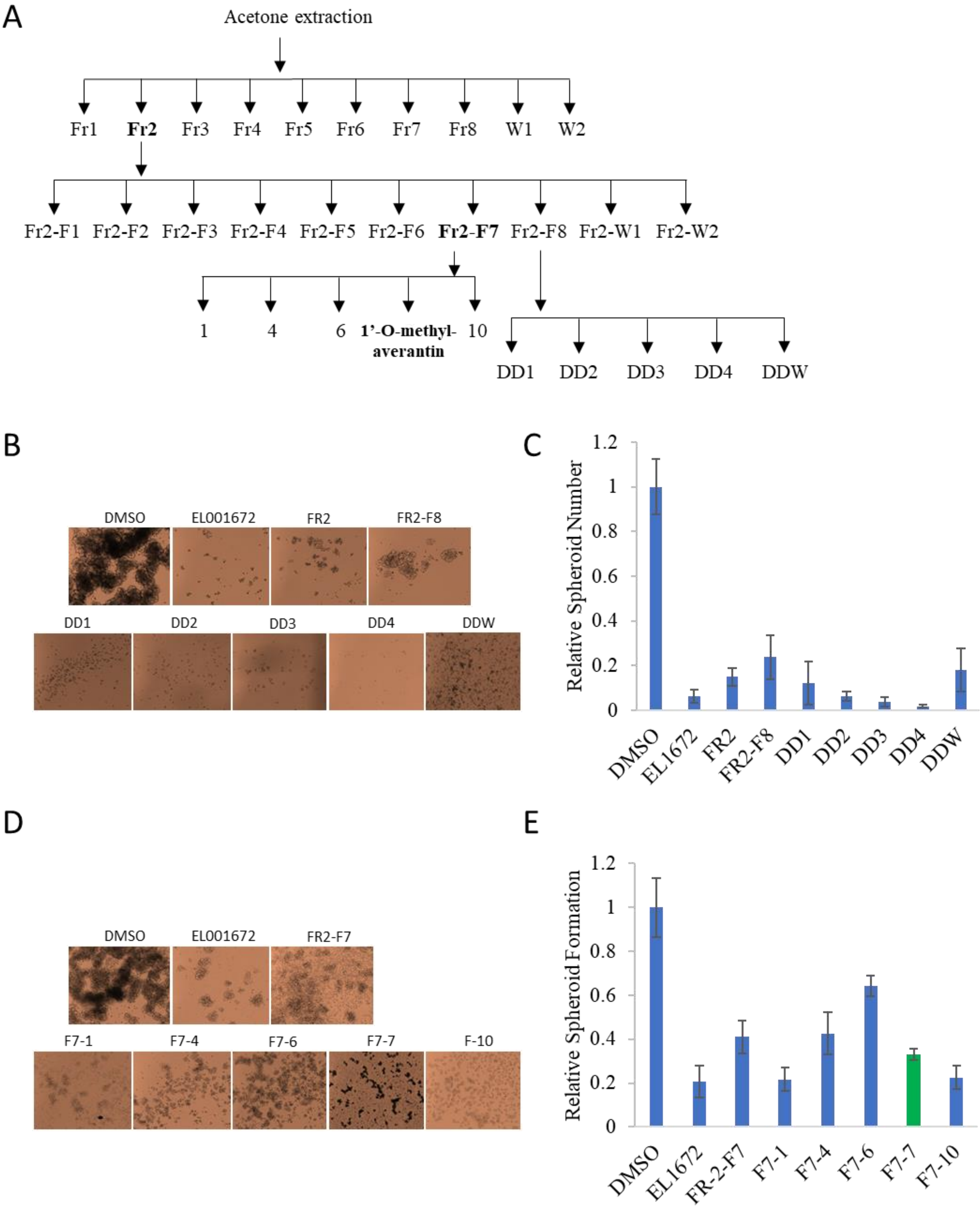

Supplementary Figure 3. (A) Flow chart of the fractionation. (B-C) Representative images of spheroid formation of CSC221 cells treated with fractions for 14 days and quantitative analysis of the number of spheroids following each treatment.

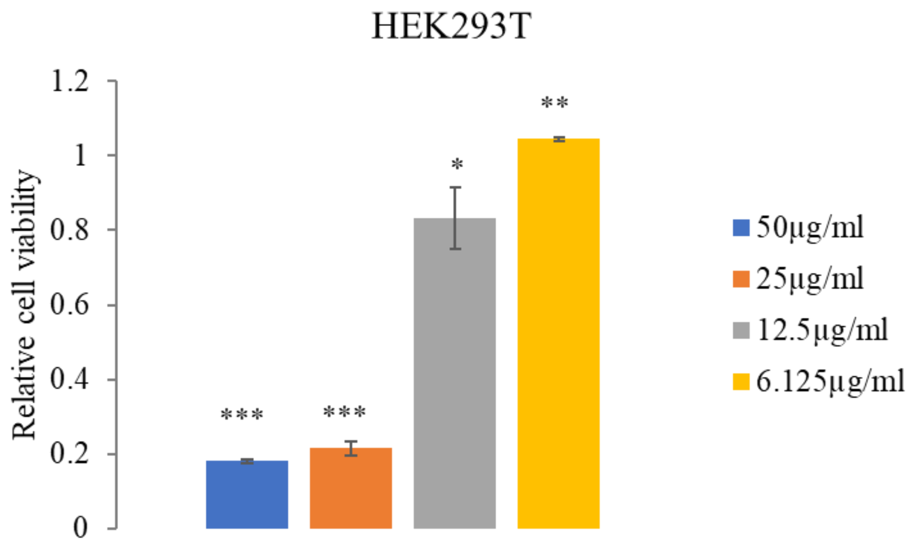

Supplementary Figure 4. Viability of HEK293T cells treated with 1'-*O*-methyl-averantin. DMSO used as a control. 50, 25, 12.5 and 6.125 μg/ml 1'-*O*-methyl averantin represent a concentration of 129.49, 64.74, 32.37 and 16.19 μM, respectively. Data represent the mean ± standard error of the mean, *p<0.05; **p<0.01; ***p< 0.001 (compared with DMSO treated cells).

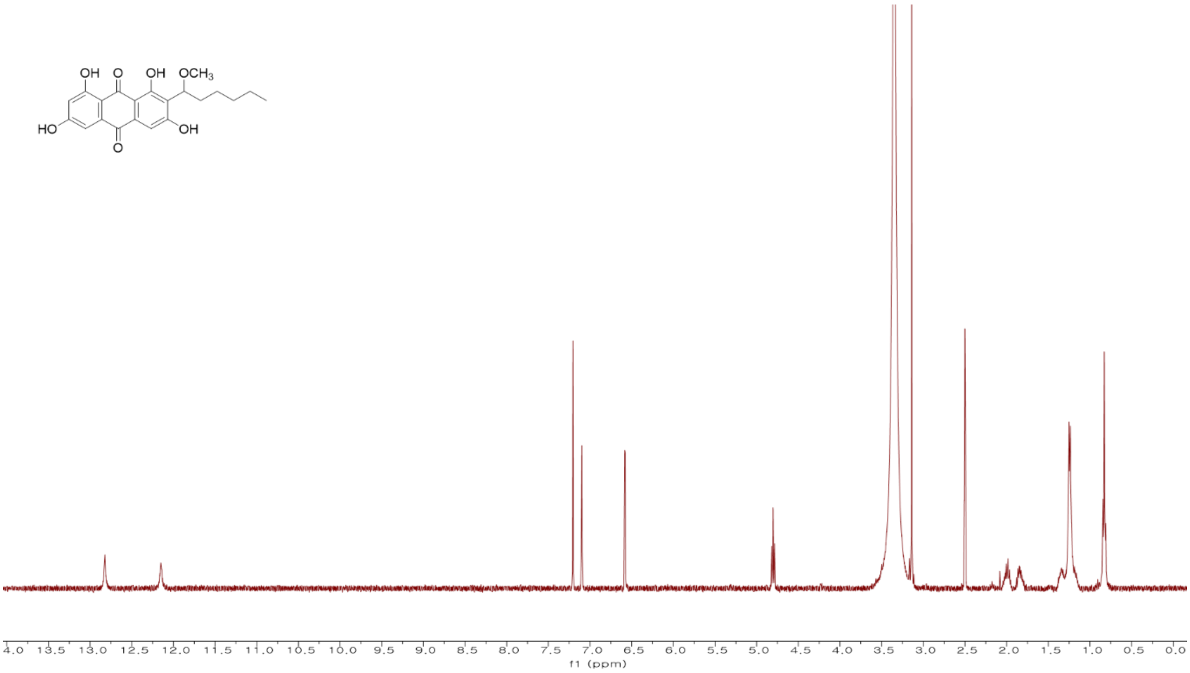

Supplementary Figure 5. ^{1}H NMR spectrum of 1’-*O*-methyl-averantin in DMSO-d_6

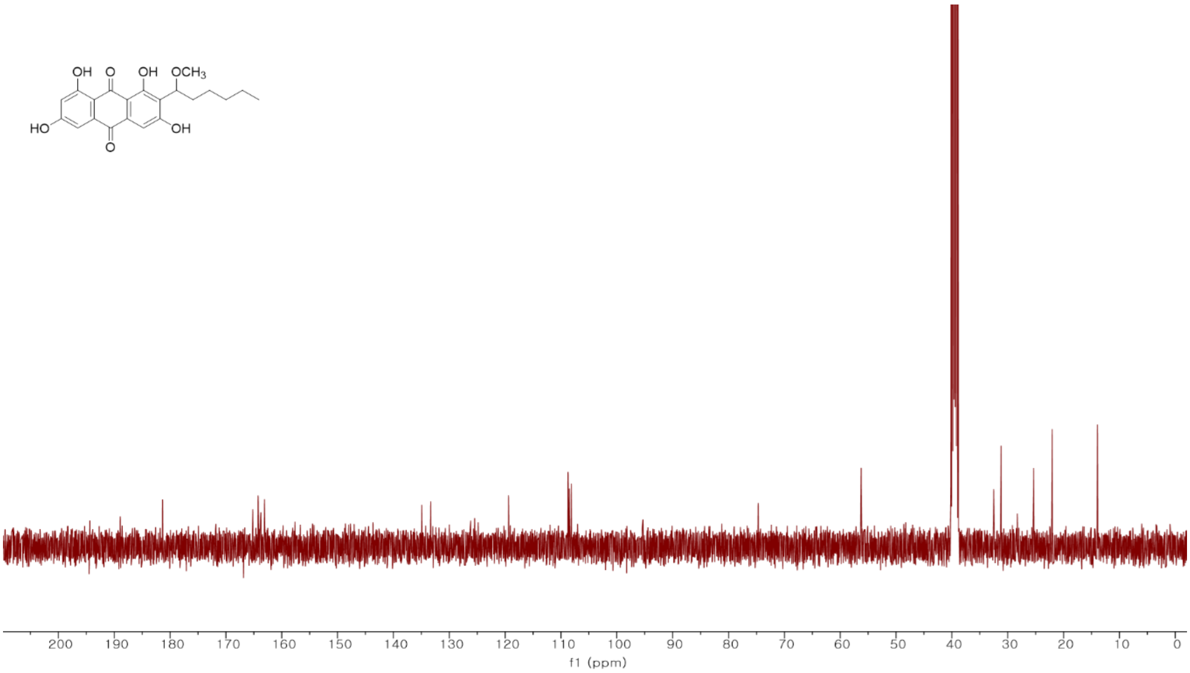

Supplementary Figure 6. ^{13}C NMR spectrum of 1’-*O*-methyl-averantin in DMSO-d_6

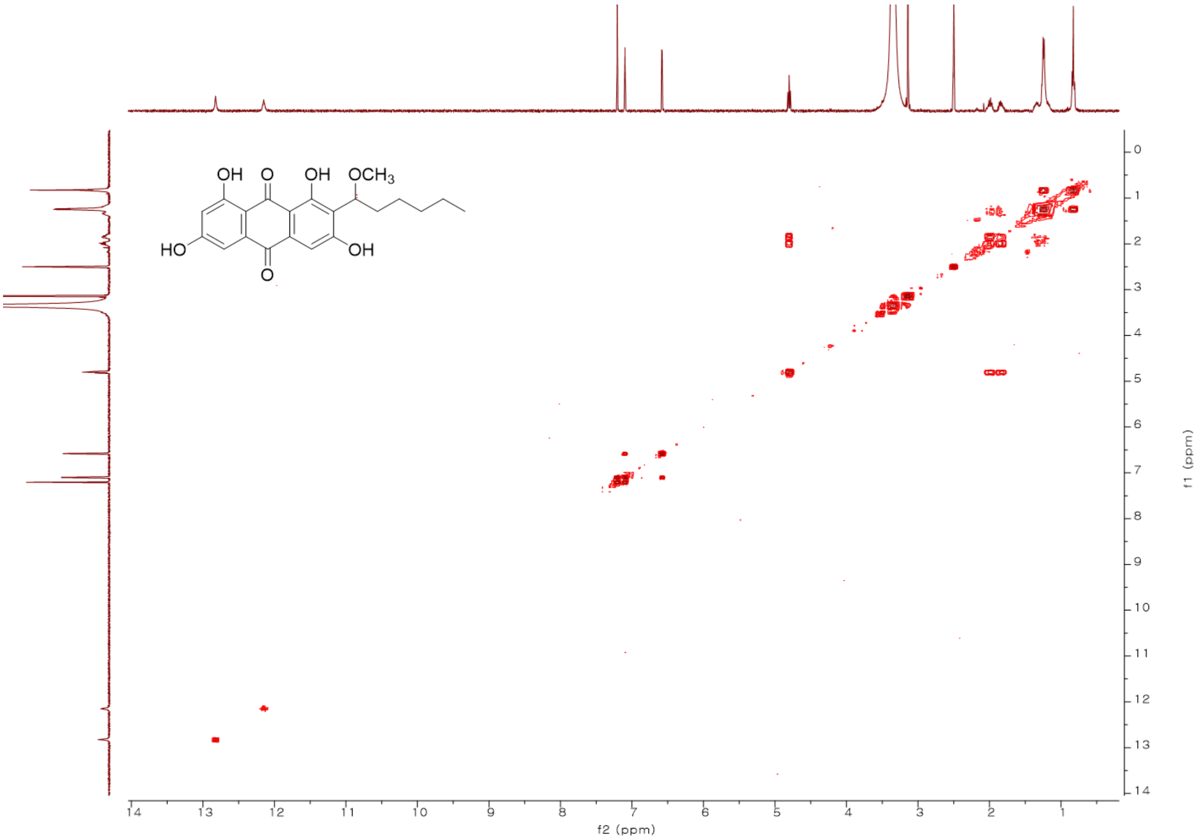

Supplementary Figure 7. COSY spectrum of 1′-*O*-methy-laverantin in DMSO-d_6

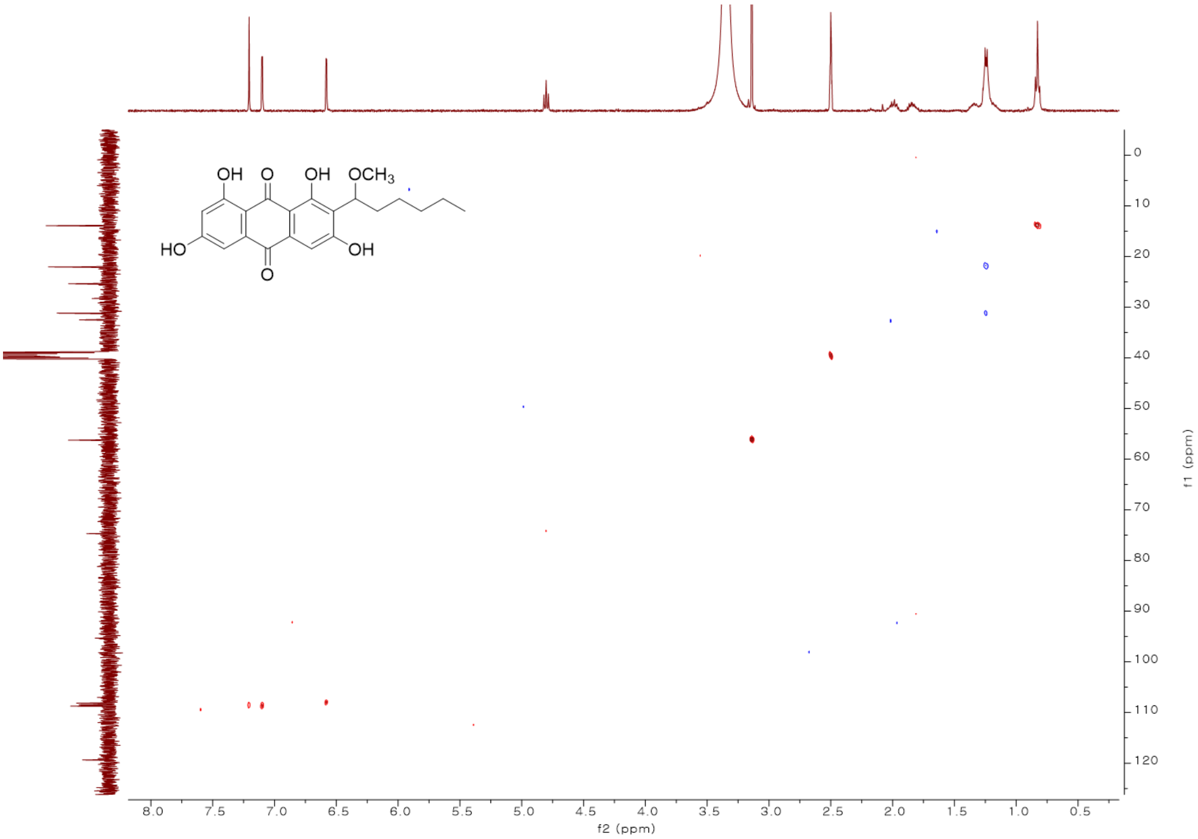

Supplementary Figure 8. HSQC spectrum of 1’-*O*-methyl-averantin in DMSO-d_6

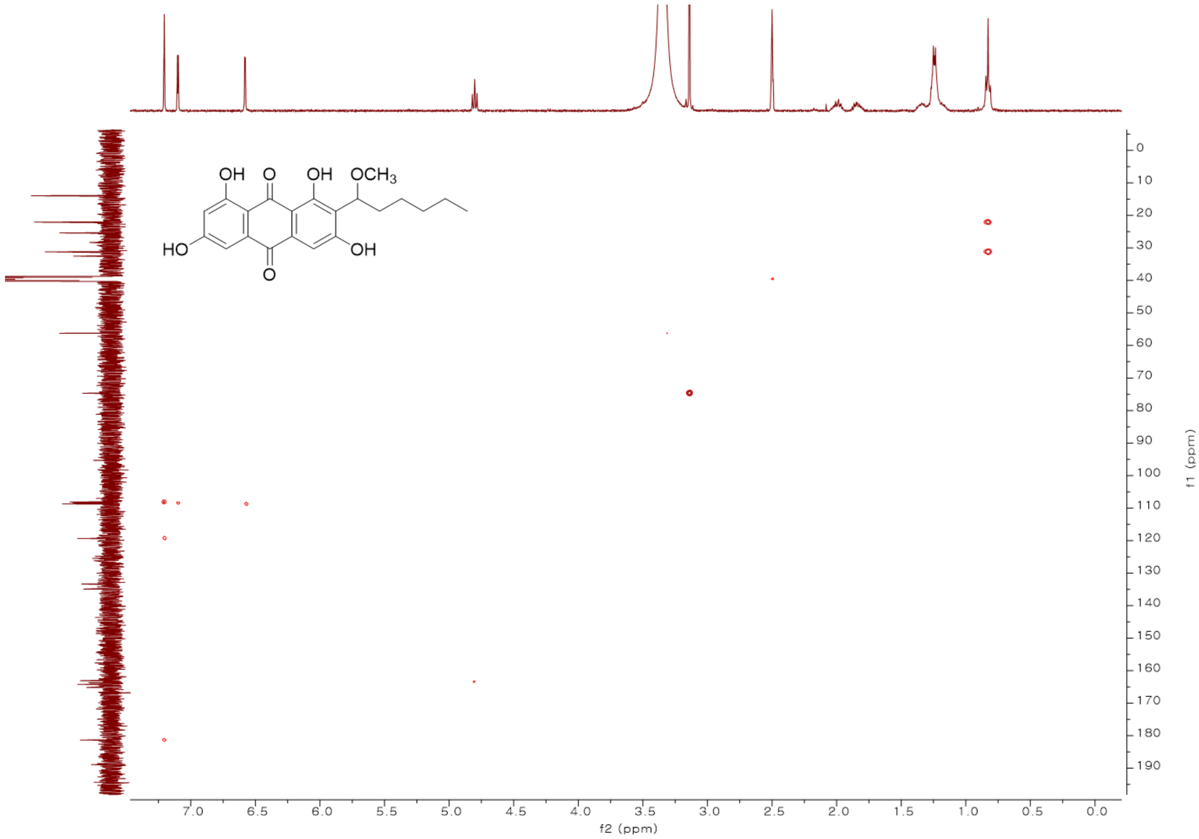

Supplementary Figure 9. HMBC spectrum of 1’-*O*-methyl-averantin in DMSO-d_6

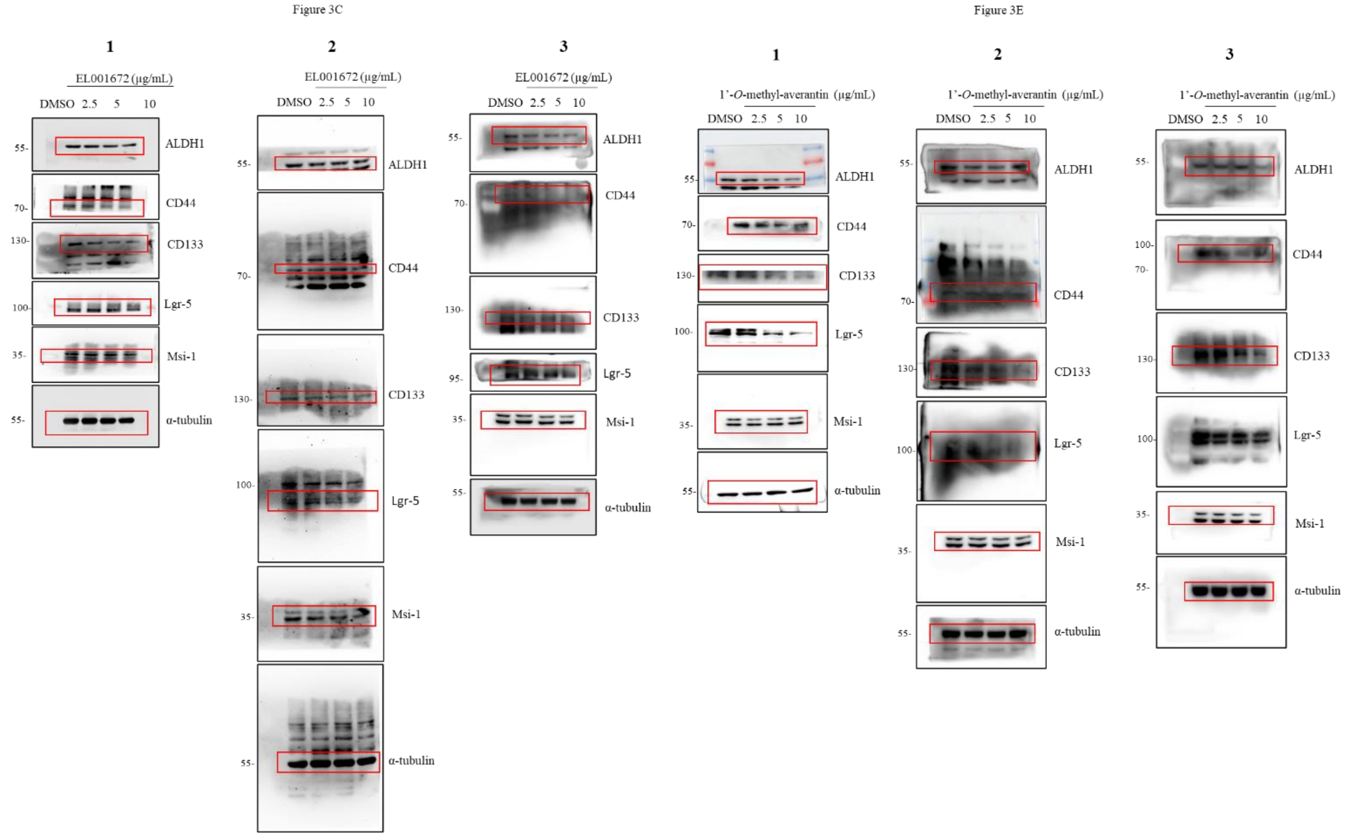

Supplementary Figure 10. Western blots in Figure 3 in the manuscript

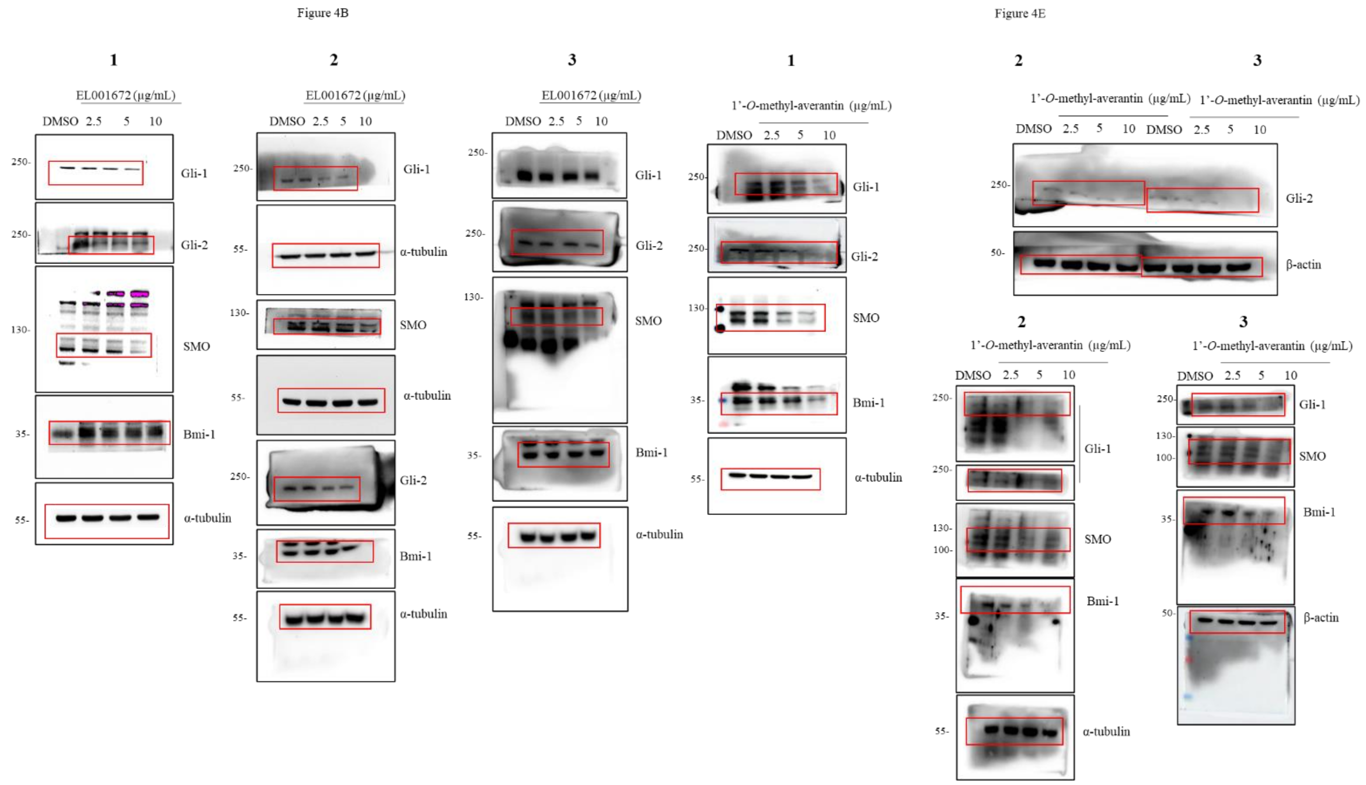

Supplementary Figure 11. Western blots in Figure 4 in the manuscript
